# Meropenem Use and Challenges in Treating Severe Infections in Pakistan Amid Antimicrobial Resistance

**DOI:** 10.1155/ipid/5531329

**Published:** 2026-07-19

**Authors:** Nasim Akhtar, Ejaz Ahmed Khan, Summiya Nizamuddin, Sana Anwar, S. H. Waqar, Sarwat Faisal, Sarmad Iqbal

**Affiliations:** ^1^ Department of Infectious Diseases, Pakistan Institute of Medical Sciences, Islamabad, Pakistan, pims.gov.pk; ^2^ Department of Pediatrics and Infectious Diseases, Shifa International Hospital, Islamabad, Pakistan, shifa.com.pk; ^3^ Department of Pathology, Shaukat Khanum Memorial Cancer Hospital and Research Centre, Lahore, Pakistan, shaukatkhanum.org.pk; ^4^ Department of Microbiology, Liaquat National Hospital & Medical College, Karachi, Pakistan; ^5^ Department of General Surgery, Pakistan Institute of Medical Sciences, Islamabad, Pakistan, pims.gov.pk; ^6^ Department of Medicine, College of Family Medicine, Karachi, Pakistan; ^7^ Department of Pharmacy Practice, Faculty of Pharmacy & Pharmaceutical Sciences, University of Karachi, Karachi, Pakistan, uok.edu.pk

**Keywords:** antimicrobial resistance, antimicrobial susceptibility, carbapenems, meropenem, pneumonia, serious infections

## Abstract

Meropenem is a broad‐spectrum carbapenem antibiotic essential for treating serious infections including urinary tract infections, pneumonia, sepsis, and meningitis. This narrative review examines meropenem resistance trends and prescribing practices for severe infections in Pakistan highlighting how inappropriate use drives local antimicrobial resistance. Relevant literature was identified through a structured search of PubMed, Scopus, Web of Science, and Google Scholar using predefined keywords. Antimicrobial resistance (AMR) is a global health crisis that complicates treatment of infections caused by multidrug‐resistant Gram‐negative pathogens, including *Acinetobacter baumannii*, *Klebsiella pneumoniae*, and *E. coli*. Pharmacokinetic studies of meropenem show extensive tissue penetration, time‐dependent bactericidal activity, and rapid renal clearance, which contribute to its effectiveness. In critically ill patients, specialized dosing strategies like continuous or extended infusions are recommended to maintain adequate drug levels. Emerging evidence also supports combination therapies (e.g., pairing meropenem with fosfomycin) to improve outcomes against carbapenem‐resistant strains. In Pakistan, these concerns are especially acute: many key pathogens show high resistance rates due to widespread antibiotic overuse and misuse. Regional surveillance indicates that *E. coli* often remains relatively sensitive to meropenem, whereas Acinetobacter species exhibit alarmingly low susceptibility. Such resistance largely stems from the spread of extended‐spectrum beta‐lactamases (ESBLs) and carbapenemase‐producing organisms, posing serious challenges to clinicians. This underscores the need for data‐driven, context‐specific strategies to guide therapy. Robust surveillance systems are needed to guide empirical therapy, and real‐time resistance monitoring can help prevent treatment failures. The review emphasizes targeted antimicrobial strategies and judicious meropenem use. Ongoing education for healthcare providers and coordinated stewardship programs at local and national levels are also essential to preserve the antibiotic’s clinical utility. Ultimately, sustaining meropenem’s effectiveness in the face of rising AMR requires combined public health efforts, including tailored dosing protocols and localized resistance management, to keep meropenem a reliable option worldwide for treating life‐threatening infections.

## 1. Introduction

Antimicrobial resistance (AMR) represents a critical global health threat, projected to cause 10 million deaths annually by 2050, largely driven by antimicrobial overuse and misuse [1, 2]. The global increase in AMR complicates the treatment of severe bacterial infections, particularly in resource‐limited areas. The incidence of multidrug‐resistant Gram‐negative bacteria (MDR‐GNBs), such as carbapenem‐resistant *Enterobacterales*, poses a significant threat to public health and restricts the treatment options for clinicians [3].

GNBs are a major cause of severe infections, including pneumonia, urinary tract infections (UTIs), sepsis, meningitis, and typhoid fever, contributing substantially to global disease burden and AMR. Gram‐negative pneumonia, commonly caused by organisms such as *Klebsiella pneumoniae* and *Pseudomonas aeruginosa*, leads to inflammation of the lung parenchyma and is often associated with higher morbidity in hospitalized and immunocompromised patients [4]. UTIs are predominantly caused by enteric Gram‐negative bacilli such as *Escherichia coli*, which can ascend the urinary tract and result in complications like pyelonephritis and bacteremia [5]. Sepsis frequently arises from Gram‐negative bloodstream infections, where endotoxin‐mediated immune responses trigger systemic inflammation, organ dysfunction, and septic shock [5]. Gram‐negative meningitis, though less common, is a serious condition often caused by organisms like *E. coli* and *Klebsiella* spp., leading to inflammation of the central nervous system with high mortality rates [6]. Additionally, typhoid fever, caused by *Salmonella enterica* serovar Typhi, represents a systemic Gram‐negative infection, with XDR strains posing significant treatment challenges due to resistance to multiple antibiotic classes [7].

The prescribing practices of meropenem indicate that it requires improved adherence to guidelines and the implementation of stewardship programs to optimize its use. The empirical prescription of meropenem is prevalent, with studies indicating that a significant proportion of prescriptions are initiated without culture results. For instance, in Oman, meropenem was prescribed empirically in 96% of the cases [8], whereas meropenem was prescribed based on culture sensitivity in only 36% of the cases in Pakistan [9].

In Pakistan, resistance to Gram‐negative infections is an established problem owing to the limited public health infrastructure, poor sanitation, and irrational use of antibiotics. The prevalence of MDR‐GNBs, especially in hospitals, is very high. Several studies have documented the widespread presence of ESBL‐producing carbapenem‐resistant *Klebsiella pneumoniae* and *E. coli*, leading to frequent treatment failures in healthcare settings [10]. Moreover*, Acinetobacter baumannii* and *Pseudomonas aeruginosa*, both well known for their resistance to multiple antibiotic classes, are responsible for a significant proportion of ventilator‐associated pneumonia (VAP), bloodstream infections, and UTIs in intensive care units across different countries [11].

Pakistan also faces significant AMR challenges, such as high resistance to common antibiotics and insufficient surveillance. Therefore, there is an urgent need for standardized monitoring and targeted actions to combat AMR effectively [12]. This review aims to provide a descriptive analysis of the role of meropenem in combating severe Gram‐negative infections, focusing on its pharmacological attributes, global evidence, and susceptibility patterns of local antibiograms in Pakistan. By analyzing the existing research on meropenem, this review highlights the current challenges posed by resistance trends, underscores the importance of precise dosing and stewardship strategies, and offers insights into the clinical utility of antibiotics as a last‐resort treatment option. This review contributes uniquely to the literature on meropenem and AMR.

## 2. Methodology: Literature Search Strategy

The approach utilized in this study was adopted to ensure comprehensive coverage of relevant evidence on meropenem use in severe infections and AMR. Literature was searched in PubMed/MEDLINE, Scopus, Web of Science, and Google Scholar up to March 2025 using predefined keywords and Boolean operators, including “meropenem,” “carbapenem resistance,” “multidrug resistant,” “extended‐spectrum beta‐lactamase,” and infection‐specific terms such as “pneumonia,” “sepsis,” “urinary tract infection,” “meningitis,” and “XDR typhoid,” combined with “Pakistan” or “global.” Inclusion criteria comprised peer‐reviewed articles, systematic reviews, surveillance reports, and guidelines focusing on meropenem pharmacokinetics (PK), pharmacodynamics (PD), susceptibility, and clinical outcomes; conference abstracts without full text and irrelevant case reports were excluded. References to selected articles were manually screened to identify additional sources.

## 3. PK and PD of Meropenem

Meropenem PK and PD are strongly influenced by the type and severity of infection. In severe infections such as sepsis, pneumonia, meningitis, complicated UTIs, and extensively drug‐resistant (XDR) typhoid fever, pathophysiological alterations significantly affect drug distribution, clearance, and target attainment [13, 14]. The inclusion of PK/PD principles in this review is therefore intended to explain variability in clinical outcomes, justify optimized dosing strategies, and highlight their relevance in high AMR settings such as Pakistan.

Meropenem is extensively distributed in body fluids and tissues, making it effective against a broad range of infections (Table 1). In critically ill patients, physiological changes significantly alter the PK parameters of drugs. For instance, the volume of distribution (Vd) increases due to fluid shifts and capillary leakage in patients with sepsis, as seen in patients with abdominal septic shock, where Vd ranged from 17.2 to 22.6 L depending on renal function [15]. Vd ranges from 0.3 to 0.4 L/kg but may vary with disease conditions, such as augmented renal clearance (ARC) [16].

**TABLE 1:** Meropenem dosage recommendations as per guidelines [13, 14].

Indication	Adult dosage	Pediatric dosage
Bacterial Meningitis	2 g/day every 8 h for 7–21 days	40 mg/kg/dose every 8 h. Maximum 2 g/dose
Hospital‐Acquired Pneumonia	1 g IV every 8 h for 7 days	10 or 20 mg/kg up to a maximum of 1 g
Infection of Skin/Subcutaneous Tissue	1 g IV every 8 h for 5–14 days	10–20 mg/kg/dose every 8 h
Sepsis	1‐2 g Q8H extended infusion	20–40 mg/kg every 8 h
Urinary Tract Infections	1 g IV every 8 h for 5–14 days	20 mg/kg q8 h; Max 1 g/dose

Meropenem, a time‐dependent β‐lactam antibiotic, can be administered via intermittent bolus infusion, extended infusion, or continuous infusion, each influencing PK and clinical outcomes. Intermittent administration produces high peak plasma concentrations followed by rapid declines, which may allow drug levels to fall below the minimum inhibitory concentration (MIC), making it suitable mainly for infections caused by highly susceptible organisms [17–19]. In contrast, extended infusion prolongs drug delivery over several hours, enhancing the fraction of time that free drug concentrations remain above the MIC (fT > MIC), thereby improving bacterial killing and potentially shortening hospital stays [20–22]. Continuous infusion maintains near‐steady plasma concentrations, optimizing fT > MIC and improving target attainment, especially in critically ill patients or infections caused by less susceptible Gram‐negative pathogens [23–25]. This approach may also reduce total drug exposure while maintaining efficacy. Although some randomized trials report similar mortality outcomes across methods, prolonged infusion strategies have been associated with improved microbiological eradication and clinical success in severe infections, particularly sepsis [26–28].

In sepsis, systemic inflammation, capillary leak, and aggressive fluid resuscitation increase the volume of distribution, while ARC accelerates drug elimination. These changes frequently lead to inadequate meropenem plasma concentrations, increasing the risk of treatment failure and resistance selection if dose optimization is not implemented [29, 30]. Critically ill patients, particularly those with ARC or septic shock, often require additional targets to avoid therapeutic failure and resistance. The recommended target for critically ill patients is 100% fT > MIC, with an even higher target of 100% fT > 4MIC in severe cases [31]. For instance, in pediatric patients, only 11% of critically ill older children achieve 100% fT > 4MIC, highlighting the challenge of attaining optimal therapeutic levels in these populations [16]. Similar findings were observed in patients with abdominal sepsis, where 1.0 g every 6 h regimens were more effective in achieving therapeutic goals [15]. A systematic review of the adult population revealed that the ideal pharmacotherapeutic dosing strategy for meropenem in critically ill individuals receiving continuous renal replacement therapy is 1 g administered every 6 h for nontrauma patients, whereas trauma patients require a dosage range of 3–4 g every 24 h [27].

In severe pneumonia, including hospital‐acquired and VAP, meropenem penetrates lung tissue and epithelial lining fluid; however, high pathogen MICs and altered pulmonary PK in critically ill patients may limit pharmacodynamic target attainment. Prolonged or continuous infusion strategies have been shown to enhance fT > MIC and improve clinical outcomes in severe lower respiratory tract infections [30, 32].

The clearance (CL) of meropenem is primarily renal, and alterations in renal function heavily influence drug clearance [15]. For complicated UTIs and urosepsis, meropenem’s predominant renal elimination results in high urinary concentrations, supporting its clinical efficacy against multidrug‐resistant uropathogens. Nonetheless, reliance on urinary accumulation alone may be insufficient in systemic infections, particularly in the presence of altered renal function, reinforcing the need for PK/PD‐guided dosing [33].

In meningitis, penetration of meropenem into cerebrospinal fluid (CSF) is enhanced by meningeal inflammation but remains variable. Sustained concentrations above the pathogen MIC are essential for bactericidal activity within the central nervous system, and high‐dose regimens may be required, especially when treating resistant organisms [34].

## 4. Pneumonia

Pneumonia is a significant global health challenge and is often linked to MDR bacteria, such as *Acinetobacter* spp. [1], P. aeruginosa [*2*], K. pneumoniae [*3*], E. coli [*4*], and Enterobacter spp. [5] representing significant contributors to the incidence of hospital‐acquired pneumonia (HAP) and VAP [35]. Carbapenem‐resistant strains, especially *K. pneumoniae* and *P. aeruginosa*, are becoming increasingly prevalent, particularly in Southeast Asia, Europe, and the Middle East [36]. This growing resistance has posed significant treatment challenges, increasing the reliance on potent carbapenems such as meropenem [37].

### 4.1. Global Evidence in Pneumonia

In recent years, evidence has shown the efficacy of meropenem, both as a monotherapy and in combination therapy. Meropenem has been shown to be noninferior to other antibiotics like cefiderocol and ceftolozane‐tazobactam in treating Gram‐negative nosocomial pneumonia. Studies indicate similar all‐cause mortality and clinical cure rates when compared with these alternatives, suggesting that meropenem is an effective treatment option [38–40].

In South Korea, Hyun et al. showed that extended infusions significantly improved the outcomes in patients with Gram‐negative pathogens, suggesting that optimizing PK through prolonged infusion may enhance the efficacy of meropenem in severe pneumonia cases [41]. A study in Iran comparing high versus standard doses of meropenem in VAP found no significant difference in clinical success rates, although high doses reduced clinical pulmonary infection scores and improved organ function [42].

A study in Italy by Cojutti et al. investigated the therapeutic drug monitoring (TDM)–guided dosing of meropenem combined with fosfomycin in a critically ill COVID‐19 patient with bacteremia and VAP caused by *K. pneumoniae* coproducing *KPC* and *OXA*‐48‐like carbapenemases in a critically ill patient. Continuous high‐dose infusions of meropenem (up to 3 g every 6 h) and fosfomycin (up to 24 g/day), guided by real‐time pharmacokinetic adjustments, resulted in bacterial clearance and clinical recovery. This underscores the importance of precise dosing for maximizing the efficacy of meropenem in life‐threatening infections [43].

### 4.2. Evidence From Pakistan

The emergence of carbapenem resistance among Gram‐negative pathogens has significantly complicated treatment strategies. Irfan et al. reported that 24% of *Escherichia coli* and 14% of *Klebsiella pneumoniae* isolates harbored the blaNDM‐1 gene, a key determinant of carbapenem resistance. The presence of this gene limits the effectiveness of last‐line antibiotics and highlights the urgent need for alternative or combination therapeutic approaches to manage MDR infections [44].

However, evidence regarding the benefit of combination therapy remains inconclusive. In contrast, another study evaluating treatment outcomes found no significant difference in 14‐day mortality between patients receiving colistin monotherapy and those treated with a colistin–meropenem combination. These findings suggest that the routine use of meropenem in combination regimens may not always confer additional survival benefit, underscoring the need for further well‐designed studies to clarify its role in MDR infections [45].

## 5. Sepsis

Sepsis affects millions of individuals annually, and mortality rates remain high despite advancements in medical care [46]. The treatment of sepsis often depends on rapid identification and effective management of causative pathogens, primarily through antimicrobial therapy. The most common causative organisms of sepsis are GNBs, such as *E. coli*, *Pseudomonas aeruginosa*, and *Klebsiella pneumoniae*, which account for a substantial proportion of healthcare‐associated infections [28, 47].

### 5.1. Global Evidence in Sepsis

The conventional dosing guidelines for various β‐lactams have been demonstrated to be insufficient in a considerable fraction of critically ill individuals. Meropenem is often used as an empirical therapy for sepsis because of its broad‐spectrum activity against both Gram‐positive and GNBs. Meropenem and imipenem are recommended for the treatment of ESBL blood stream infections (BSIs) in critically ill patients with sepsis [48].

A meta‐analysis by Chen et al. found that continuous infusion of meropenem in septic patients significantly reduced mortality by 34%, improved clinical cure rates, and enhanced microbiological eradication compared to intermittent infusion. This suggests that maintaining constant plasma concentrations of meropenem above the MIC is crucial for bacterial clearance, particularly in critically ill patients [28]. In USA, Ahmed et al. compared extended and intermittent infusions in critically ill patients. The extended infusion group had lower ICU mortality (19% vs. 37%) and better clinical response rates (83% vs. 46%) than the intermittent infusion group did. The extended infusion protocol improved drug plasma level control and reduced the risk of subtherapeutic exposure, which is crucial for patients with sepsis with altered renal clearance and fluid shifts [21].

In Spain, extended infusion regimens of meropenem enhance bactericidal activity, especially against resistant strains [49]. In China, Cao et al. showed that extended infusion significantly improved clinical effectiveness and microbial clearance compared with short‐term infusion (STI). Notably, very low birth weight infants had a 75.6% effectiveness rate with extended infusion versus 56.6% with STI, underscoring the need for tailored dosing strategies for vulnerable populations such as neonates [50]. In India, Kothekar et al. analyzed the effectiveness of a 3‐h extended infusion of meropenem (1 g every 8 h) in adult patients with severe sepsis and septic shock. The research demonstrated that extended infusion was effective in maintaining plasma concentrations above the MIC for sensitive Gram‐negative pathogens. However, it was also revealed that standard dosing regimens were inadequate against pathogens requiring higher exposure, suggesting that higher doses or more frequent dosing intervals may be necessary for optimal outcomes in certain patient populations [51].

Critically ill patients with sepsis often present with significant physiological changes, including increased renal clearance, which can lead to suboptimal antibiotic exposure if standard‐dosing regimens are used. In Belgium, Gijsen et al. found that conventional meropenem dosing often failed to meet PK/PD targets in nearly 60% of critically ill septic patients with augmented renal function. This highlights the need for individualized dosing and recommended dose escalation or continuous infusion to ensure adequate drug exposure and to prevent therapeutic failure [52].

### 5.2. Evidence From Pakistan

Sepsis poses a substantial burden on Pakistan’s healthcare system, particularly as a low‐ and middle‐income country (LMIC) with a high prevalence of infectious diseases. Respiratory tract infections and UTIs are the leading sources of sepsis, with GNBs identified as the predominant causative pathogens. Inadequate adherence to standardized sepsis management guidelines further contributes to poor clinical outcomes and elevated mortality rates. Reported mortality rates vary, with one study documenting sepsis‐associated mortality at 37%, while another reported an overall mortality rate of 40.7% [53]. Microbiological analyses consistently highlight the predominance of Gram‐negative organisms in septic patients. Abbasi et al. identified *Escherichia coli* and *Pseudomonas aeruginosa* as the most frequently isolated pathogens from blood cultures. In this study, meropenem demonstrated 100% sensitivity against *Pseudomonas aeruginosa* [47].

However, contrasting findings have been reported. Hashmi et al. observed a markedly lower sensitivity rate of 28.6% for meropenem against Gram‐negative organisms, including *Acinetobacter baumannii* and *Klebsiella pneumoniae*, both of which exhibited high resistance to multiple antibiotic classes. Despite this reduced sensitivity, meropenem remains a critical therapeutic option in neonatal sepsis due to the limited availability of effective alternatives [54].

In pediatric populations, meropenem continues to play a pivotal role, particularly in infections caused by MDR organisms. Iqbal et al. [55] reported that GNBs, especially *E. coli* and *Klebsiella pneumoniae*, account for the majority of sepsis cases. In their study, meropenem showed high efficacy, with a susceptibility rate of 83.3%. The authors emphasized that carbapenems, including meropenem, should be reserved for severe or refractory cases to mitigate the risk of escalating AMR [55].

Neonatal sepsis remains a critical concern, with *Klebsiella* species identified as the most common causative agents. Anwar et al. reported that *Klebsiella* spp. accounted for 58.3% of positive blood cultures. The associated mortality rate was notably high at 39.3%, particularly among preterm and low‐birth‐weight infants. Although meropenem remains one of the most effective treatment options, emerging carbapenem resistance—reported in approximately 50%–60% of isolates—poses a significant threat to its future clinical utility in neonatal care settings [56].

## 6. UTIs

Urinary tract diseases are among the most frequently encountered infectious diseases and usually require antibiotics. They can be encountered at any age in both the sexes. The age‐standardized incidence rate (ASIR) of UTIs among females was observed to be 3.59 times greater than that recorded for males, with the peak incidence noted within the 30–34 age demographic range [57]. Furthermore, recurrent UTIs are more common in women, with nearly 20%–30% of women who have had one UTI experiencing recurrence within 6 months.

### 6.1. Global Evidence in UTIs

UTIs are among the most common bacterial infections worldwide, affecting millions of people annually, particularly women. *E. coli* is the predominant etiological agent of community‐acquired UTIs, with 62.5% of isolated strains exhibiting profiles indicative of multidrug resistance [58]. Carbapenems are preferred when resistance or toxicity prevents the use of trimethoprim–sulfamethoxazole (TMP–SMX) or fluoroquinolones, or when a patient is critically ill with pyelonephritis or complicated UTIs with ESBL‐producing *Enterobacterales* (ESBL‐E) [59]. In the 1990s, meropenem demonstrated superior antimicrobial activity against *E. coli* among clinical isolates, and pharmacodynamic studies indicated that it significantly reduced viable bacterial counts [60, 61]. Meropenem has also demonstrated efficacy in pyelonephritis, systemic infections, and pneumonia caused by E. coli [62, 63]. Meropenem resistance was first reported in the USA in 2005, with the identification of the blaKPC‐3 gene, followed by a case involving ESBL in a UTI isolate in 2009 [64, 65].

A comparative study of meropenem against different antibiotics in the elderly population with UTIs regarding the effect of ESBL‐producing *E*. *coli* was conducted. Meropenem showed greater efficacy in severe UTI cases than most of the antibiotics assessed, including fosfomycin/minocycline, rifampicin/sulfamethoxazole–trimethoprim, levofloxacin, and cefoperazone/sulbactam [66].

Albiero et al. demonstrated the effectiveness of meropenem against ESBL‐producing *E. coli* against UTIs in vitro. Combining meropenem with fosfomycin enhanced its antimicrobial activity against metallo‐beta‐lactamase (MBL)‐producing strains, significantly lowering the MIC and restoring its efficacy against resistant strains. With a reported clinical cure rate of 87%, this study suggests the use of meropenem in conjunction with other agents to treat MDR *E. coli* [67]. Meropenem‐vaborbactam, a combination therapy, has been shown to be noninferior to plazomicin in clinical trials, suggesting that meropenem is effective. However, combination therapies may enhance treatment outcomes against resistant infections [68].

In a Japanese study examining the efficacy of meropenem against carbapenem‐resistant *E. coli* strains, researchers identified meropenem resistance as a critical marker for defining carbapenem‐resistant Enterobacteriaceae (CRE). This finding is particularly important given that meropenem often remains effective in cases where imipenem and other carbapenems fail [69].

### 6.2. Evidence From Pakistan

*Escherichia coli* remains the most prevalent uropathogen in Pakistan, followed by *Klebsiella pneumoniae*, both of which have demonstrated notable susceptibility to meropenem in acute and chronic UTIs [70]. Although meropenem has shown strong efficacy in the treatment of complicated UTIs (cUTIs), the emergence of AMR is an increasing concern. Several studies have reported moderate resistance rates, particularly in regions with high levels of antibiotic consumption. Mohydin et al. emphasized the importance of antimicrobial stewardship programs to reduce the overuse of broad‐spectrum antibiotics, including meropenem, as a strategy to curb the development and spread of resistant strains [71]. Comparative susceptibility data further contextualize meropenem’s role in UTI management. Waseem et al. reported that *E. coli* isolates from urine samples exhibited a 95.5% susceptibility rate to meropenem, slightly lower than fosfomycin (99.25%) and nitrofurantoin (97%). While meropenem remains highly effective, fosfomycin and nitrofurantoin may represent more cost‐effective treatment options, particularly for infections caused by extended‐spectrum β‐lactamase (ESBL)‐producing *E. coli* [72].

MDR *E. coli*, a major contributor to UTI burden, has shown high resistance rates to commonly prescribed antibiotics such as ciprofloxacin and cotrimoxazole. In contrast, meropenem continues to demonstrate high sensitivity, making it a reliable therapeutic option in cases where first‐line agents fail [70]. Supporting this, Azhar et al. reported an 89.1% sensitivity rate of meropenem in MDR *E. coli* isolates from UTI cases in Faisalabad, Pakistan [73].

Nosocomial UTIs, frequently associated with prolonged hospitalization and invasive procedures, present additional therapeutic challenges due to the high prevalence of MDR organisms. Shabbir et al. demonstrated the high efficacy of meropenem in hospital‐acquired UTIs, with *E. coli* and *Citrobacter* spp. showing sensitivity rates of 97.8% and 93.8%, respectively [74]. However, emerging resistance trends are concerning. Khan et al. reported increasing resistance in *Klebsiella pneumoniae*, with rates reaching 28%, indicating a gradual decline in meropenem effectiveness against certain pathogens [75]. These findings underscore the urgent need for robust antimicrobial stewardship and the development of alternative therapeutic strategies to combat resistant uropathogens in Pakistan.

## 7. Meningitis

Meropenem has emerged as an effective treatment option for bacterial meningitis owing to its broad‐spectrum activity and utility in treating infections caused by MDR organisms. Meningeal inflammation in bacterial meningitis can enhance the penetration of meropenem and other β‐lactams into the CSF by increasing blood–brain barrier (BBB) permeability [76].

### 7.1. Global Evidence in Meningitis

For cases involving resistant organisms, studies have demonstrated that high‐dose meropenem administered as prolonged or continuous infusion can achieve superior CSF concentrations. A case report of a patient with meningitis caused by carbapenemase‐producing *Acinetobacter baumannii* when treated with a significantly higher than standard dose of meropenem (15 g per day) resulted in CSF concentrations up to 64 mg/L, sufficient to exceed the pathogen’s MIC by a substantial margin [77]. Another patient with meningitis caused by a resistant *Enterobacter cloacae* strain was successfully treated with high‐dose, prolonged infusion (2 g thrice daily, infusion ≥ 3 h) of meropenem and both intravenous and intraventricular amikacin [78]. This combination effectively eradicated the infection despite the pathogen’s high meropenem MIC (≥ 16 mg/L), highlighting the role of meropenem in treating severe, resistant CNS infections. These approaches have shown promise in managing pathogens with high resistance levels and provide a path for conducting randomized clinical trials on high‐dose prolonged infusion of meropenem to generate evidence that can be generalized to a broader population.

The Turkish Ephesus study demonstrated comparable success to that of alternative regimens involving ceftazidime and cefepime. Despite the high prevalence of MDR organisms in healthcare settings, the combination of meropenem and vancomycin achieved clinical success rates and survival outcomes similar to other standard therapies, including cefepime plus vancomycin and ceftazidime plus Vancomycin [79]. A multicenter study on healthcare‐associated meningitis (HCAM) found that meropenem with vancomycin had similar results [79].

A Swedish study found no significant difference in 30‐day and 90‐day mortality rates between meropenem and a cefotaxime–ampicillin combination for community‐acquired acute bacterial meningitis (ABM) [80]. The 30‐day mortality rate was 3.2% for cefotaxime–ampicillin and 3.6% for meropenem, suggesting that meropenem offers equivalent outcomes while covering a broader range of pathogens, including resistant pathogens.

Children with meningitis, especially those who are critically ill or have ARC, often require adjustments for meropenem. Studies have highlighted the importance of continuous or prolonged infusion to maintain effective CSF levels [31]. Pediatric dosing must also account for factors, such as age‐related PK and differences in BBB permeability. Individualized dosing regimens are beneficial for maintaining meropenem above the MIC of the pathogen and for optimizing its bactericidal effects [76]. A dose of 40 mg/kg every 8 h is recommended for improved clinical outcomes, and meropenem is generally well tolerated in children, even at higher doses, with a low incidence of adverse effects, making it a preferable choice in cases requiring aggressive therapy [81, 82].

In China, Jin et al. highlighted that the standard dosing regimen might be insufficient in such cases, necessitating higher or continuous dosing to maintain the required therapeutic levels. These findings reinforce the importance of TDM in adjusting doses for both adult and pediatric patients with ARC or other conditions that affect renal clearance, ensuring that meropenem remains above the MIC for effective bacterial eradication [83].

A Swedish national registry study found that meropenem monotherapy was noninferior to standard cefotaxime and ampicillin combination therapy for treating ABM, with comparable mortality rates. The broad‐spectrum and favorable safety profile of meropenem make it a feasible first‐line treatment, especially in adult cases with less severe pathogen resistance. [84].

### 7.2. Evidence From Pakistan

Bacterial meningitis remains a serious public health concern in Pakistan, associated with high morbidity and mortality. A cross‐sectional study conducted in Quetta identified the causative pathogens, including *Streptococcus pneumoniae* (14.5%), *Staphylococcus aureus* (6.8%), *Neisseria meningitidis* (5.7%), *Haemophilus influenzae* (2.5%), *Escherichia coli* (4.5%), and *Klebsiella pneumoniae* (1.9%). Antimicrobial susceptibility testing in this study demonstrated high efficacy of meropenem against several pathogens, with sensitivity rates of 92.5% for *E. coli*, 58.8% for *K. pneumoniae*, and 100% for both *H. influenzae* and *N. meningitidis* [85].

Combination antibiotic therapy has also been explored to improve clinical outcomes. Ayub et al. reported the successful use of meropenem in combination with vancomycin in 15% of pediatric cases of ABM. This regimen is particularly beneficial in targeting a broad spectrum of pathogens, including Gram‐negative organisms such as *E. coli* and *Acinetobacter* spp., which are commonly associated with hospital‐acquired infections [86]. Despite these therapeutic strategies, outcomes remain poor in many cases. A multicenter study in Pakistan reported a mortality rate of 66% among patients with bacterial meningitis, underscoring the urgent need for optimized treatment protocols and strengthened antimicrobial stewardship [87].

Further evidence from CSF culture and susceptibility profiling highlights the role of GNBs in meningitis. *E. coli* has been identified as a common pathogen, demonstrating approximately 60% susceptibility to meropenem. Although lower than previously reported rates, this finding still supports the use of meropenem as a valuable treatment option, particularly in cases where first‐line therapies fail or resistance limits alternative choices [88]. In specific clinical scenarios, meropenem has shown effectiveness as a second‐line agent. For instance, in cases of *Salmonella* meningitis unresponsive to initial therapy with ceftriaxone and vancomycin, switching to meropenem resulted in significant clinical improvement. This highlights its importance in managing refractory or resistant infections [89].

## 8. Use of Meropenem in Severe XDR Typhoid

The emergence of MDR and extensively drug‐resistant (XDR) *Salmonella typhi* in Pakistan has raised significant concerns regarding typhoid fever management. This public health issue is exacerbated by the widespread use of antibiotics in the region, which has contributed to the increasing resistance of *S. typhi* to common antimicrobial agents. MDR strains of *S. typhi* are resistant to first‐line treatments such as ampicillin, chloramphenicol, and trimethoprim–sulfamethoxazole, whereas XDR strains, first identified in Pakistan in 2016, are resistant to these drugs, as well as third‐generation cephalosporins and fluoroquinolones [90, 91]. These developments have severely limited treatment options, leading to the need for more expensive and less widely available antibiotics such as meropenem and azithromycin [92, 93].

Recent studies have highlighted that the XDR *S. typhi* is a significant threat to Pakistan, with outbreaks affecting both children and adults. Between 2016 and 2019, more than 5000 cases of XDR typhoid were reported in Karachi, and the infection continued to spread to other regions [91, 94]. Azithromycin is recommended at a dose of 10 mg/kg per day orally for 7–14 days, with a maximum dose of 500 mg/day for adults, whereas meropenem is used in cases where azithromycin alone does not yield sufficient results, with a dose of 20–40 mg/kg every 8 h for children and 1–2 g every 8 h for adults administered intravenously [92].

Meropenem has been found to be highly effective against XDR S. typhi, a strain that is resistant to multiple classes of antibiotics, including third‐generation cephalosporins, fluoroquinolones, and older agents such as ampicillin and chloramphenicol. Studies indicate that meropenem can significantly reduce the time to defervescence (the period when fever subsides), a critical indicator of successful treatment [93, 95]. In particular, a combination of meropenem and azithromycin is recommended when meropenem alone does not show rapid effects [93]. A case of carbapenem‐resistant S. typhi infection harboring the NDM‐5 gene in a child was treated with high‐dose meropenem administered over an extended infusion in combination with colistin, highlighting the difficulty in treating common endemic infections [96].

Meropenem has shown similar efficacy in different settings, with studies from Karachi and Lahore demonstrating good clinical outcomes in patients with XDR typhoid, with fever resolution typically occurring within 6–7 days of initiating treatment [97]. However, the high cost of meropenem remains a limitation, particularly in resource‐limited settings, making it less accessible for some populations.

## 9. Overview of Meropenem Susceptibility in Pakistan

**FIGURE 1:**
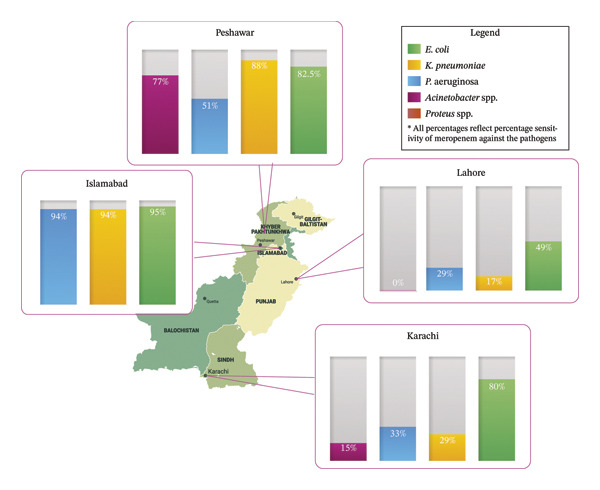
Antimicrobial susceptibility (% sensitivity) of meropenem against different pathogens from Pakistan.

Antimicrobial sensitivity patterns of meropenem in different cities in Pakistan revealed significant regional differences. In Peshawar, the susceptibility rates were as follows: *Pseudomonas aeruginosa*, 51%; *E. coli*, 82.5%; *Klebsiella pneumoniae*, 88%; and *Acinetobacter spp*., 77% [98].In Lahore, Ikram et al. reported *P. aeruginosa* at 29%, *E. coli* at 49%, *K. pneumoniae* at 17%, and *Acinetobacter spp*. at 0% [99]. Islamabad reported higher susceptibility, with *P. aeruginosa* at 94%, *E. coli* at 95%, and *K. pneumoniae* at 94% [100]. Karachi contained *P. aeruginosa* (33%), *E. coli* (80%), *K. pneumoniae* (29%), and *Acinetobacter spp*. (15%) [101] (Figure 1).

These variations underscore the critical need for localized antimicrobial stewardship in Pakistan. The notably low susceptibility of *Acinetobacter spp*. in Lahore (0%) and Karachi (15%) is particularly concerning, as infections caused by meropenem‐resistant bacteria are associated with higher treatment failure rates, prolonged hospital stays, increased healthcare costs, and elevated mortality rates [102]. The emergence and spread of meropenem‐resistant strains have significantly limited their effectiveness, thereby posing a critical threat to clinical practice [102]. To address these challenges, clinicians must utilize updated antibiograms to evaluate bacterial susceptibility patterns and rationalize empirical antibiotic therapy [103].

### 9.1. In‐Patient Data

The marked variability in susceptibility rates among pathogens and institutions underscores the importance of localized antibiograms in tailoring treatment strategies. For instance, *Acinetobacter spp*., a known MDR organism, exhibits alarmingly low susceptibility rates, especially at Shifa International Hospital, Islamabad (14%), necessitating stringent infection control protocols and consideration of alternative therapeutic options. Conversely, the high susceptibility of *P. mirabilis* across all centers (> 95%) suggests that meropenem remains an effective choice for treating infections caused by this pathogen. The relatively high susceptibility rates for *E. coli* and *P*. *aeruginosa* (> 70% at most centers) are promising, but the moderate rates for *K*. *pneumoniae* (54%–76%) and *Enterobacter* spp. (77%–89%) highlight the need for cautious use to prevent further resistance development [104] (Table 2).

**TABLE 2:** In‐patient meropenem susceptibility against different pathogens in tertiary care hospitals of Pakistan.

Organism	Meropenem susceptibility data (in‐patient)			
	SKMHRC	LNH	PIMS	SIH
*Acinetobacter spp*.	38%	—	23%	14%
*E. coli*	73%	—	83%	81%
*Enterobacter spp*.	89%	—	77%	—
*Klebsiella pneumoniae*	76%	—	62%	54%
*Pseudomonas aeruginosa*	83%	—	67%	63%
*Proteus mirabilis*	99%	100%	95%	98%

*Note:* SKMHRC—Shaukat Khanum Memorial Hospital & Research Centre Lahore, LNH—Liaquat National Hospital Karachi, PIMS—Pakistan Institute of Medical Sciences Islamabad, SIH—Shifa International Hospital Islamabad.

### 9.2. Outpatient Data

The particularly low susceptibility rates of *Acinetobacter* spp. (23%–31.4%) highlight the limited utility of meropenem for treating infections caused by this pathogen in outpatient settings, requiring alternative therapeutic options and enhanced infection prevention measures. In contrast, *P*. *mirabilis* consistently demonstrated high susceptibility (> 94% across all centers), supporting meropenem as a reliable treatment option for infections involving this organism. The moderate susceptibility rates of *K*. *pneumoniae* (38.8%–71%) and *Enterobacter* spp. (54.7%–73%) suggest the need for cautious use of meropenem, especially in regions with lower efficacy, to mitigate the risk of resistance development. The relatively higher susceptibility of *E*. *coli* to SKMHRC and SIH (90%) than to AKUH (50.5%) underscores the importance of localized antibiograms in guiding empirical therapy. Additionally, the susceptibility of *P*. *aeruginosa* (63%–73.8%) suggests that meropenem remains a viable option for managing these infections, despite institutional variability [104] (Table 3).

**TABLE 3:** Outpatient meropenem susceptibility against different pathogens in tertiary care hospitals of Pakistan.

Organism	Meropenem susceptibility data (outpatient)			
	AKUH	SKMHRC	LNH	SIH
*Acinetobacter* spp.	31.4%	23%	—	30%
*E. coli*	50.5%	90%	—	90%
*Enterobacter* spp.	54.7%	73%	—	—
*Klebsiella pneumoniae*	38.8%	70%	—	71%
*Pseudomonas aeruginosa*	73.8%	63%	—	64%
*Proteus mirabilis*	94%	97%	100%	97%

*Note:* AKUH—Aga Khan University Hospital Karachi, SKMHRC—Shaukat Khanum Memorial Hospital & Research Centre Lahore, LNH—Liaquat National Hospital Karachi, SIH—Shifa International Hospital Islamabad.

## 10. Limitations

This narrative review is based on published literature, much of which comprises retrospective studies, single‐center data, or surveillance reports with heterogeneous methodologies. Local antimicrobial susceptibility patterns reported here may be subject to sampling bias, variation in laboratory standards, and incomplete geographical representation within Pakistan. In addition, reliance on secondary data limits the ability to account for unreported variables, temporal changes in resistance trends, and potential publication bias. These factors should be considered when interpreting the findings and extrapolating them to broader clinical settings.

## 11. Conclusion

Meropenem remains crucial for severe Gram‐negative infections, including MDR cases, but rising resistance, especially in *K. pneumoniae* and *Acinetobacter spp*., demands robust stewardship, optimized dosing, and active surveillance. In Pakistan, gaps include the lack of randomized trials on combination therapies, limited research on resistance‐breaking strategies, and inconsistent multicenter data. Addressing these through targeted clinical studies and improved monitoring will be essential to preserve meropenem’s effectiveness in resource‐limited settings and ensure its continued role in combating life‐threatening infections.

**Nomenclature**
ABM: Acute bacterial meningitis
AKUH: Aga Khan University Hospital (Karachi)
AMR: Antimicrobial resistance
ARC: Augmented renal clearance
ASIR: Age standardized incidence rate
BBB: Blood–brain barrier
BSI: Blood stream infection(s)
cUTI: Complicated urinary tract infection
CI: Continuous infusion (antibiotic dosing)
CL: Clearance (pharmacokinetics)
CrCl: Creatinine clearance
CNS: Central nervous system
CRE: Carbapenem‐resistant enterobacterales
CSF: Cerebrospinal fluid
ESBL: Extended spectrum β lactamase
ESBL E: ESBL producing enterobacterales
fT > MIC: Fraction of the dosing interval at which the free drug remains > MIC
GNB: Gram‐negative bacteria
HAP: Hospital acquired pneumonia
HCAM: Healthcare‐associated meningitis
ICU: Intensive care unit
KPC: Klebsiella pneumoniae carbapenemase
LMIC: Low‐ and middle‐income countries
LNH: Liaquat National Hospital (Karachi)
MDR: Multidrug resistant
MDR GNB: Multidrug‐resistant Gram‐negative bacteria
MIC: Minimum inhibitory concentration
MBL: Metallo β lactamase
NDM: New Delhi metallo‐β‐lactamases (e.g., NDM 1, NDM 5)
OXA: OXA‐type carbapenemase (e.g., OXA 48)
PD: Pharmacodynamics
PK: Pharmacokinetics
PK/PD: Pharmacokinetic/pharmacodynamic (relationship)
PIMS: Pakistan Institute of Medical Sciences (Islamabad)
SIH: Shifa International Hospital (Islamabad)
SKMHRC: Shaukat Khanum Memorial Hospital & Research Centre (Lahore)
STI: Short‐term infusion (≤ 30 min bolus)
TDM: Therapeutic drug monitoring
TMP SMX: Trimethoprim–sulfamethoxazole
UTI/UTIs: Urinary tract infection(s)
VAP: Ventilator‐associated pneumonia
Vd: Volume of distribution
XDR: Extensively drug resistant

## Funding

This study did not receive any external funding.

## Conflicts of Interest

The authors declare no conflicts of interest.

## Data Availability

Data sharing is not applicable to this article as no datasets were generated or analyzed during the current study.
